# Improving efficiency of semitransparent organic solar cells by constructing semitransparent microcavity

**DOI:** 10.1038/s41598-023-36488-4

**Published:** 2023-06-12

**Authors:** Elmira Annabi Milani, Mina Piralaee, Asghar Asgari

**Affiliations:** 1 Faculty of Physics, University of Tabriz, Tabriz, 51665-163 Iran; 2 Photonics Devices Research Group, Research Institute of Applied Physics and Astronomy, University of Tabriz, Tabriz, 51665-163 Iran; 3 School of Electrical, Electronic, and Computer Engineering, University of Western Australia, Crawley, WA 6009 Australia

**Keywords:** Physics, Energy infrastructure

## Abstract

Semitransparent organic solar cells have become attractive recently because of their photon harvesting in the near-infrared and ultraviolet range and passing in the visible light region. Semitransparent organic solar cells with Glass/MoO3/Ag/MoO3/PBDB-T:ITIC/TiO2/Ag/PML/1DPCs structure have been studied in this work and the effects microcavity with 1-dimensional photonic crystals (1DPCs) on the solar cell performance such as the power conversion efficiency, the average visible transmittance, Light utilization efficiency (LUE), the color coordinates in the CIE color space, and CIE LAB are investigated. The analytical calculation including the density of exactions and their displacement is used to model the devices. The model shows that the presence of microcavity can improve the power conversion efficiency by about %17 in comparison with the absence of microcavity. Although the transmission is decreasing slightly, microcavity does not change the color coordinates much. The device can transmit high-quality light with a near-white sensation to the human eye.

## Introduction

Organic solar cells (OSCs) have attracted much attention due to their advantages such as low cost, easy fabrication, flexibility, and recently their potential applications for Semi-transparent Solar Cells (ST-SCs), although their stability is challenging^1–7^. ST-SCs, combining the benefits of light-to-electricity conversion and light transparency, have emerged as one of the most prominent energy harvesting technologies. These technologies can be used for Agri-voltaic (as the roof of greenhouses), and windows for buildings. The performance of ST-SCs depending on their applications is generally determines by their capability to convert the incident light into electricity while allowing transmitted light through the device.

ST-SCs require not only a transparent active layer but also both electrical contacts, electron transport layer (ETL), and hole transport layer (HTL) to be transparent in a wide spectral range (from IR to UV) along with an efficient collection of photo-generated charge carriers. For front electrodes, transparent conductive oxide^8^, thin metal film^9–11^, the conductive polymer^12,13^, graphene^14,15^, and nanotube films^16^ have been previously used and also for back electrodes mostly organic active bulk heterojunction layers are used^17^. However, using a transparent active layer and electrodes decreases the device efficiency^9^. To overcome this problem, a method is coating one-dimensional photonic crystals (1DPCs) on the top electrode of ST-SC^17–22^. Although the fabrication of the 1DPCs with a few to a dozen layers is a challenge and make additional fabrication cost, the advantage of this light trapping structure is considerable since the 1DPCs can increase the efficiency of the ST-SCs by reflecting all the photons with energies less than the photon bandgaps (PBGs) for re-absorption in the active layer.

It should be mentioned that the active layer of the ST-SCs cannot completely absorb the reflected light by the 1DPCs since the thickness of the active layer is so thin. For conventional opaque OSCs, some methods have been previously developed and applied for light trapping. The methods are based on optical spacers, surface plasmons, and optical microcavity^23–30^. Using optical microcavity in combination with optical spacers can confine a large number of photons within the device and increase light absorption in the active layer^25,30^. To increase the absorption of reflected photons from 1DPC in the active layer of ST-SCs, considering the improvement in absorption is usually accompanied by a reduction in transparency, it is even more challenging to develop light-trapping structures which can improve photon absorption without reducing the transparency of semitransparent devices.

This paper aims to develop a light-trapping structure to enhance photon absorption in ST-SCs using a semitransparent microcavity. For this purpose, the active layer is sandwiched between the MoO_3_/Ag/MoO_3_ multilayer electrode and the Ag electrode which is capped by 1DPCs. The proposed structure without 1DPC has been previously studied by our group in terms of different structural parameters to improve efficiency and transparency, and an efficiency of about 4% with 45% transparency is reported. In this paper, the theoretical calculations based on Transfer-Matrix Method (TMM)^31,32^ show that the semitransparent microcavity can improve the power conversion efficiency of ST-SCs. The effects of the variable pairs of 1DPCs on the power conversion efficiency of ST-SCs and their transparency are also investigated.

Another commonly reported figure of merit for ST-SCs is light utilization efficiency (LUE). Taking into account both PCE and AVT, a direct comparison of LUE values can hold viable information in contrast to the direct comparison of the AVT values without knowledge of the PCE^33^.

Achieving widespread adoption of semi-transparent organic solar cell technology requires combined optimization of PCE and AVT. While electronic displays require AVT > 80% (LUE > 5%), architectural tinted glass requirements typically start closer to 50%. PCE values of 5–10% (LUE > 2.5%) are required in BIPV (Building Integrated PV) applications to reduce electricity costs. However, 2–5% PCE (LUE > 1.5%) is sufficient for low-power mobile electronic devices. TPVs with similar PCE but lower AVT (LUE > 1%) can self-power smart windows or complement passive window coatings^34,35^. Recently, a high-order LUE transparent solar cell (LUE = 5.46%) with an efficiency of 9.1% and an AVT of 60% has been reported. Another reported work with LUE = 2.2% concerns a wavelength-selective DSSC device (PCE = 6.1%, AVT = 36%) with a low-cost diphenylamine-based dye and a highly transparent iodine-free electrolyte. Among inorganic translucent devices, CIGS cells are the ones with the best performance of LUE = 1.3%. Other inorganic semi-transparent devices have been demonstrated with Cu_2_ZnSn(S, Se)_4_ and Sb_2_S_3_-based solar cells with still low performance (LUE < 1%)^36^.

### Device structure and theoretical model

The modeled ST-SCs consist of Glass/MoO_3_(I)/Ag/MoO_3_(II)/Active layer (PBDB-T:ITIC)/TiO_2_/Ag/LiF/1DPCs structures. Where MoO_3_/Ag/MoO_3_ acts as the transparent top electrode, and the inner MoO_3_ layer (30 nm) acts as the HTL. Sol–gel processed ZnO layer is used as the ETL, and ITO is the transparent bottom electrode. MoO_3_(I) /Ag also acts as the input antireflection layer with a thickness assumed to be 10/6 nm, and Ag/LiF/1DPCs act as the output antireflection layer with thickness set as 10 nm, and different numbers of pairs of 1DPC to construct semitransparent microcavity and then improve the optical performance of the ST-SCs.

**Figure 1:**
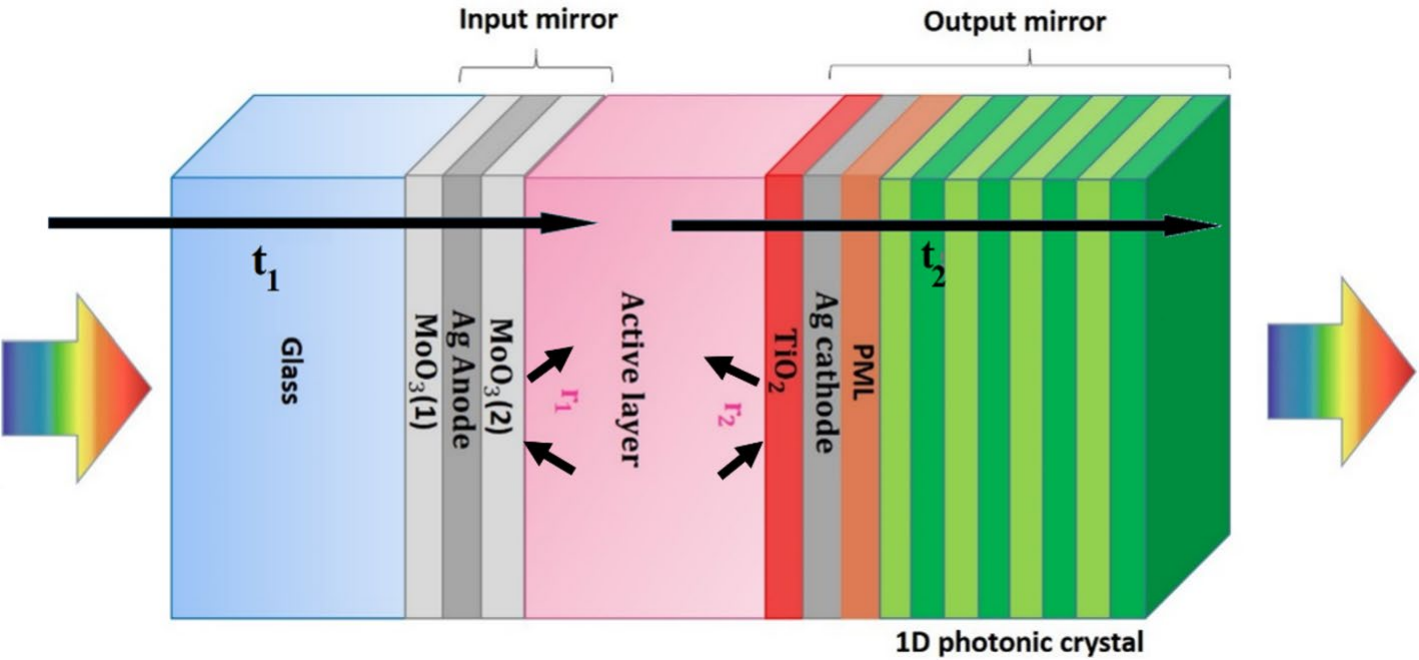
The structure of modeled 1DPCs-based ST-SCs with microcavity structure; t_1_ and t_2_ denote the transmission coefficient of input and output mirrors; and r_1_ and r_2_ denote the reflection coefficient of input and output mirrors, respectively.

The thickness of the electron transport layer (TiO_2_) is set to 10 nm and the thickness of the active layer (PBDB-T: ITIC) is assumed to be 100 nm. The phase matching layer (PML), LiF film is sandwiched between the Ag electrodes and set to 84 nm. The 1DPCs are assumed to be composed of different pairs of WO_3_/LiF, as is illuminated in Fig. 1. The layer thicknesses of the WO_3_ and the LiF are determined by Eq. 1^37^:

1 $${n}_{{WO}_{3}}{d}_{{WO}_{3}}{=n}_{LiF}{d}_{LiF}=\frac{{\lambda }_{0}}{4}$$

where n_WO3_ and n_LiF_ denote the refractive index of the WO_3_ and LiF layers, and d_WO3_ and d_LiF_ denote the thickness of WO_3_ and LiF layers, respectively; λ is the center wavelength of the photonic bandgap of the 1DPCs. A schematic view of the structure of glass/MoO_3_(I)/Ag/MoO_3_(II)/active layer/TiO_2_/Ag/PML/1DPCs layers which according to thin film optics^30^ acts as a microcavity is shown in Fig. 1.

**Figure 2:**
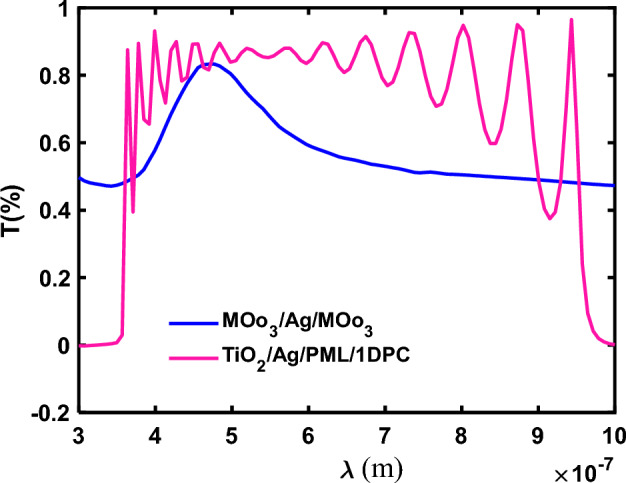
The transmission of the input mirror (MoO_3_(I)/Ag/MoO_3_(II)) and output mirror (TiO_2_/Ag/PML/1DPCs) of the microcavity.

The transmission of the input (MoO3(I)/Ag/MoO3(II)) and output (TiO2/Ag/PML/1DPCs) mirrors of the constructed microcavity for the passband wavelength of the 1DPCs (300 nm–1000 nm) is shown in Fig. 2. As shown in the figure, the related passband confirms the device transparency after using the microcavity. Knowing the reflectance (R1, and R2) and transmission (T1 and T2) of the cavity mirrors, the transmission of the device can be calculated using thin film optics and is expressed as^38^:

2 $$T\left(\lambda \right)=T\frac{{T}_{1}{T}_{2}{e}^{-2\beta }}{{(1-{{(R}_{1}{R}_{2}{e}^{-2\beta })}^{1/2})}^{2}}$$

where $\beta =2\pi kd/\uplambda$ and k denote the extinction coefficient of the active layer.

To model the device transparency, the transmittance of all individual layers, and all together, are calculated using the TMM method^25^. It should be reminded that the transparency properties of a device are determined by both average visible transmittance, AVT, and by transmittance characteristics in the visible light wavelength range (370–740 nm), taking into account the photopic response of the human eye *V*(λ).

To calculate the device’s performance parameters such as short-circuit current (Jsc), open-circuit voltage (V_oc_), fill-factor (FF), and power conversion efficiency (PCE), we used the drift–diffusion model, where, in addition to considering the density of excitons, their displacement is taken into account. The calculated AVT value is extensively explained in our previous publications^39^. We have used Eq. (3) to consider the effects of PCE and AVT simultaneously (LUE)^33^:

3 $$LUE=PCE\times AVT$$

To qualify the implementation in practical applications such as architectural window glass and mobile surfaces, aesthetics are just as significant as PCE for TPV devices. Aesthetic quality can be quantitatively estimated from three main figures of merit: the AVT, color rendering index (CRI), and the CIELAB color coordinates (a*, b*). The calculation of AVT, CRI, and color coordinates requires the transmittance spectrum of the OTPV as the input data. The CIELAB is a device-independent, 3-dimensional color space that enables precise measurement and comparison of all perceivable colors using three color values.

In this color space, numerical differences between values correspond to the amount of change humans see between colors, which was defined by the International Commission on Illumination (abbreviated CIE) in 1976.

It expresses color as three values: L* for perceptual lightness and a* and b* for the four unique colors of human vision: magenta, green, blue, and yellow. In this paper, we have reported the CIELAB color space parameter set (a*, b*), which indicates the relative color concerning a reference illumination source^34^.

The acceptable range of CIELAB color space parameters for various transparent devices is − 5 < a* < 1 and − 5 < b* < 5. The 3D approximately uniform, color space is produced by plotting in rectangular coordinates, L *, a*, b*, quantities defined as the Eqs. (4a)–(4c).

4a $$L^{*} = 116f(Y/Y_{n} ) - 16$$

4b $$a^{*} = 500[f(X/X_{n} ) - f(Y/Y_{n} )] \,$$

4c $$b^{*} = 200[f(Y/Y_{n} ) - f(Z/Z_{n} )]$$

With,

5 $$\left\{ \begin{gathered} f(t) = \sqrt[3]{t} \, t > (24/116)^{3} \, \hfill \\ f(t) = (841/108)(t) + 16/116 \, t \le (24/116)^{3} \, \hfill \\ \end{gathered} \right.$$

where $X$, $Y$,$Z$ are the tristimulus values of the test object color stimulus considered and $X_{n}$, $Y_{n}$, $Z_{n}$ are the tristimulus values of a specified white object color tristimulus. Generally, the specified white object color stimulus should be light reflected from a perfect reflecting diffuser illuminated by the same light source as the test object^36^.

## Result and discussion

To calculate the performance parameters of the ST-SCs, two different structures (Device A and B) have been taken into account and the obtained results are compared and the key parameters are shown in Table 1. Device A (a ST-SC with a microcavity) has a structure of Glass/MoO_3_(10 nm)/Ag(6)/MoO_3_(30 nm)/active layer (100 nm)/TiO_2_(10 nm)/Ag/PML/1DPCs (with 8 pairs of WO_3_/LiF photonic crystal) and The Conventional Device B (No cavity) has a structure of Glass//MoO_3_(10 nm)/Ag(6 nm)/MoO_3_(30 nm)/active layer (100 nm)/TiO_2_(10 nm)/Ag(10 nm).

**Table 1:** Key characteristics for semitransparent OSCs with/without the microcavity.

Device	Type	J_sc max_ (mA/cm^2^)	PCE_max_ (%)	AVT (%)
A	With microcavity	12.01	8.72	22.24
B	No-Microcavity	10.55	7.52	25.90

**Figure 3:**
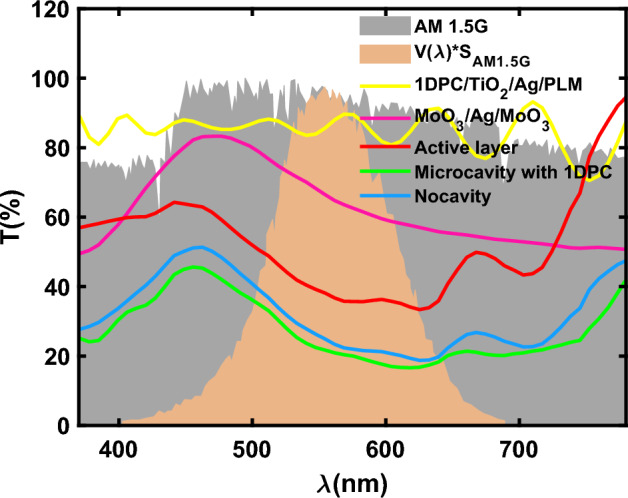
The calculated transmission spectrum of the active layer (100 nm), without photonic crystal, (blue line) with photonic crystal (green line), under AM1.5 spectral radiation and optical sensitivity *V*(λ). *SAM*1.5 (λ).

For this purpose, the transmission spectrum of devices A and B including the active layer, and the bottom and the top reflecting layers (MoO_3_(I)/Ag/MoO_3_(II) as input mirror and TiO_2_/Ag/PML/1DPCs as output mirror) in the visible medium region are calculated and shown in Fig. 3. The amount of transmission in device A is reduced for all wavelengths, but this reduction is less for the eye sensitive area (orange color in Fig. 3). Reduction of the transmission spectrum means that the absorption spectrum for Device A is improved and this improvement is attributed to the resonance effects of the semitransparent microcavity.

**Figure 4:**
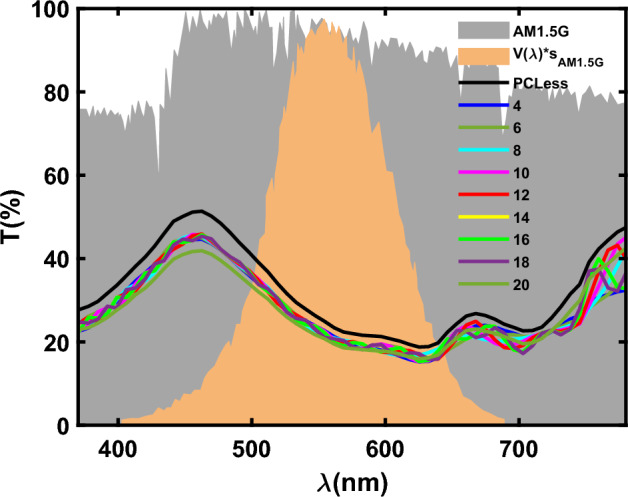
The transmission spectrum of the solar cell without micro-cavities and micro-cavities from 4 to 20 pairs of layers.

**Figure 5:**
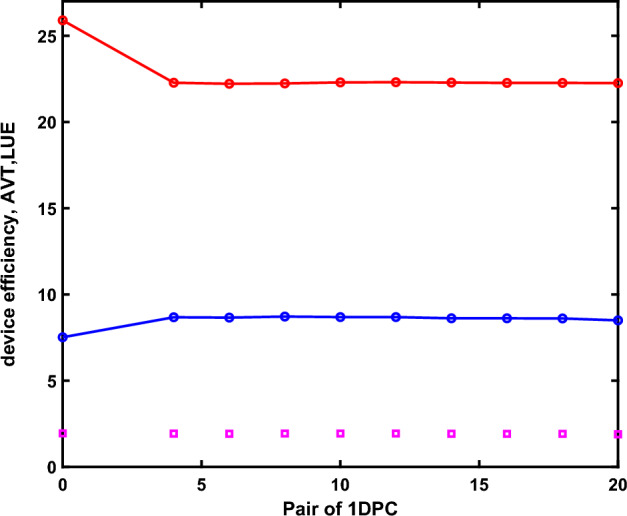
The AVT (%), PCE (%), and LUE (%) of the devices as a function of the number of 1DPC pair layers.

In the next step, we investigated the effects of the number of photonic crystal layers on cells’ efficiency and transparency. We have calculated the transmission of the devices with the structure including 4 to 20 pairs layers of photonic crystal and related results are shown in Fig. 4. The results show that the number of photonic crystal layers does not have a great impact on the overall transparency and therefore on the performance parameters of the solar cell, although the behavior of transmission for different wavelengths is slightly different from a different number of photonics crystal layers. The effects of these pairs of layers on the PCE, AVT, and LUE of ST-SCs are investigated and depicted in Fig. 5, and Table 2.

**Table 2:** The AVT, PCE, and LUE of the semitransparent OSCs with/without the microcavity.

	AVT(%)	PCE (%)	LUE (%)
No- microcavity	25.90	7.52	1.94
4 pairs of microcavity	22.28	8.68	1.93
6 pairs of microcavity	22.22	8.66	1.92
8 pairs of microcavity	22.24	8.72	1.93
10 pairs of microcavity	22.30	8.69	1.93
12 pairs of microcavity	22.31	8.69	1.93
14 pairs of microcavity	22.29	8.62	1.92
16 pairs of microcavity	22.27	8.62	1.91
18 pairs of microcavity	22.27	8.61	1.91
20 pairs of microcavity	22.26	8.50	1.89

Calculations show that the photonic crystal with 8 pairs of layers in the structure can change the value of PCE and AVT, so It’s found that PCE improved from 7.52 to 8.72% and AVT decreased from 25.9.19 to 22.24%. By increasing the number of layers, there is not much change in PCE and AVT. Therefore, PCE can be increased with microcavity even with a minimum number of pairs of layers (at least two pairs). Also, from Fig. 5 and Table 2, it could be found that there isn’t a significant difference between the reported values of LUE for different pairs of 1DPCs, however, there is an optimum situation in the case of 8- 12 pairs of 1DPCs.

**Figure 6:**
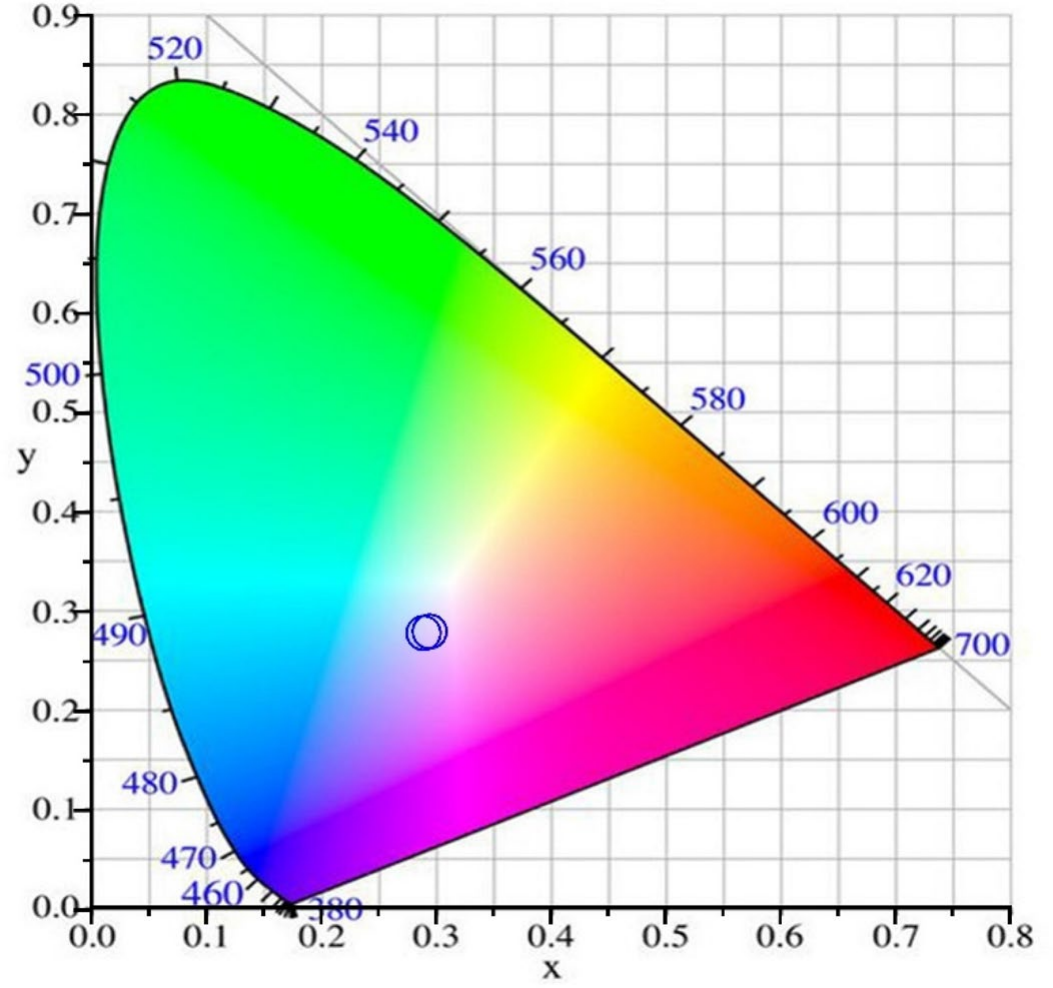
Representation of the color coordinate (x, y) of the ST-OSC with (without) microcavity, under standard D65 illumination light source on the CIE1931 color space.

The CIE color space, including the coordinates of ST-OSC for both devices (with and without microcavity), is shown in Fig. 6. The color coordinates of both devices are very close to each other and located beside the color point or so-called "white dot" in the CIE chromaticity diagram. Using microcavity changes the color coordinates slightly, but the device can transmit high-quality light with a near-white sensation to the human eye with a very small changing the original color of an object.

It should be pointed out that the PCE and AVT data in this paper are calculated using the model reported in our previous article^37^. As usual, the practical device PCE and AVT may be smaller than the calculated PCE and AVT data proposed in this paper. But, optical modeling in this paper can provide in-depth knowledge on how to apply semitransparent microcavity to simultaneously improve the photon absorption and transparency of the semitransparent OSCs. Predictive and descriptive research results obtained from these researches are considered very helpful for the design, fabrication, and experimental study of 1DPCs-based semitransparent OSC devices.

**Figure 7:**
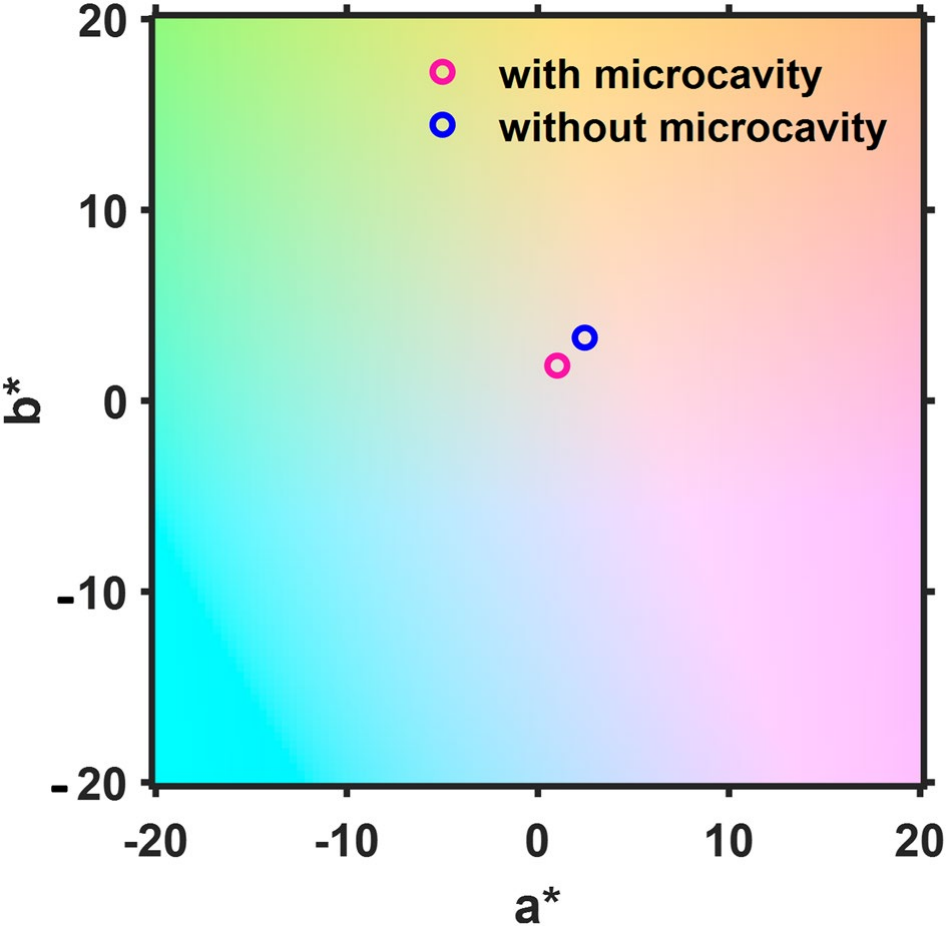
The CIELAB color space parameter set (a*, b*) of the OTPV in two cases: with and without using microcavity.

Figure 7 represents the CIE LAB color space parameter set (a*, b*) of the ST-OSCs in two cases: with and without using microcavity. As expected, similar to the CIE color coordinate space diagram, for both cases, the values of a* and b* are nearby to each other located close to the white point.

**Figure 8:**
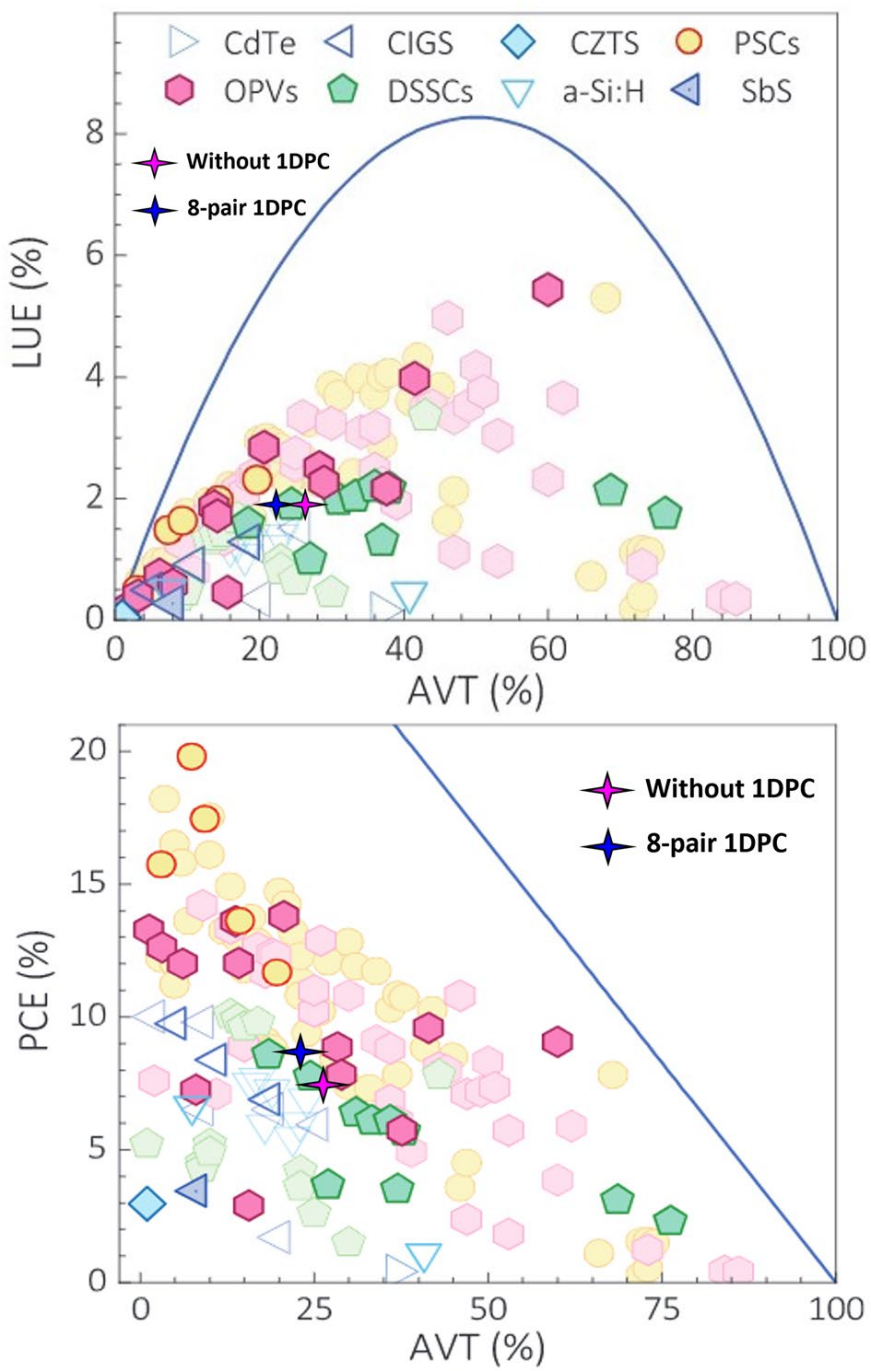
The obtained results in the cases of without and with 8-pairs of 1DPCs in comparisons with the best performing transparent and semitransparent PVs: (**a**) LUE versus AVT and (**b**) The PCE as a function of AVT. (Experimental data are taken from^36^).

In Fig. 8a, we have compared the LUE versus AVT for our cases, without and with 8-pairs of 1DPCs, with some reported experimental data. As shown in the figure, the LUE does not change, and there is a very small change in AVT. Also, the calculated LUE for the optimum structure (8- pairs layers of 1D-PC), is in acceptable coincidence with the similar ST-OPVs. Figure 8b, shows the PCE versus AVT for our cases, without and with 8-pairs of 1DPCs, in comparison with some reported experimental data. As depicted in the figure, the PCE is changed^36^.

## Conclusion

In this paper, the light trapping structure based on semitransparent microcavity is constructed for ST-SCs by sandwiching an active layer between the (MoO_3_/Ag/ MoO_3_) multilayer electrode and a thin Ag electrode capped by 1DPCs. We have investigated the effects of different pairs of 1-dimensional photonic crystals (1DPCs) on the ST-SC’s properties such as the PCE, AVT, LUE, the color coordinates in the CIE color space, and CIE LAB. As a result, the presence of microcavity can improve the power conversion efficiency by about %16 in comparison with the absence of microcavity. Although the transmission is decreasing slightly (14% decreasing), using microcavity does not change the color coordinates much and the device can transmit high-quality light with near-white sensation to the human eye.

## Data Availability

The datasets used and/or analysed during the current study available from the corresponding author on reasonable request.

## References

[1] Tai Q, Yan F (2017). Emerging semitransparent solar cells: Materials and device design. Adv. Mater..

[2] Piralaee M, Asgari A (2021). Bimetallic core–shell nanoparticles to improve the absorption of P3HT: PCBM organic solar cell. Appl. Opt..

[3] Emmott CJM, Röhr JA, Campoy-Quiles M, Kirchartz T, Urbina A, Ekins-Daukes NJ, Nelson J (2015). Organic photovoltaic greenhouses: a unique application for semi-transparent PV?. Energy Environ. Sci..

[4] Wong YQ, Meng HF, Wong HY, Tan CS, Wu CY, Tsai PT, Chang CY, Horng SF, Zan HW (2017). Efficient semitransparent organic solar cells with good color perception and good color rendering by blade coating. Org. Electron..

[5] Cui Y, Yang CY, Yao HF, Zhu J, Wang YM, Jia GX, Gao F, Hou JH (2017). Efficient semitransparent organic solar cells with tunable color enabled by an ultralowbandgap nonfullerene acceptor. Adv. Mater..

[6] dos Reis Benatto GA, Roth B, Corazza M, Søndergaard RR, Gevorgyan SA, Jørgensen M, Krebs FC (2016). Roll-to-roll printed silver nanowires for increased stability of flexible ITO-free organic solar cell modules. Nanoscale.

[7] Ren M, Sweelssen J, Grossiord N, Gorter H, Eggenhuisen TM, Andriessen R (2012). Inkjet printing technology for OPV applications. J. Imaging Sci. Technol..

[8] Colsmann A, Puetz A, Bauer A, Hanisch J, Ahlswede E, Lemmer U (2011). Efficient semi-transparent organic solar cells with good transparency color perception and rendering properties. Adv. Energy Mater..

[9] Chueh CC, Chien SC, Yip HL, Salinas JF, Li CZ, Chen KS, Chen FC, Chen WC, Jen AK-Y (2013). Toward high-performance semi-transparent polymer solar cells: optimization of ultra-thin light absorbing layer and transparent cathode architecture. Adv. Energy Mater..

[10] Chen KS, Salinas JF, Yip HL, Huo L, Hou J, Jen AK-Y (2012). Semi-transparent polymer solar cells with 6% PCE, 25% average visible transmittance and a color rendering index close to 100 for power generating window applications. Energy Environ. Sci..

[11] Li FM, Ruan SP, Xu Y, Meng FX, Wang JL, Chen WY, Shen L (2011). Semitransparent inverted polymer solar cells using MoO3/Ag/WO3 as highly transparent anodes. Sol. Energy Mater. Sol. Cells.

[12] Tang Z, George Z, Ma Z, Bergqvist J, Tvingsted K, Vandewal K, Wang E, Andersson LM, Andersson MR, Zhang F, Inganäs O (2012). Semi-transparent tandem organic solar cells with 90% internal quantum efficiency. Adv. Energy Mater..

[13] Czolk J, Puetz A, Kutsarov D, Reinhard M, Lemmer U, Colsmann A (2013). Inverted semi-transparent polymer solar cells with transparency color rendering indices approaching 100. Adv. Energy Mater..

[14] Lee YY, Tu KH, Yu CC, Li SS, Hwang JY, Lin CC, Chen KH, Chen LC, Chen HL, Chen CW (2011). Top laminated graphene electrode in a semitransparent polymer solar cell by simultaneous thermal annealing/releasing method. ACS Nano.

[15] Liu ZK, You P, Liu SH, Yan F (2015). Neutral-color semitransparent organic solar cells with all-graphene electrodes. ACS Nano.

[16] Kim YH, Muller-Meskamp L, Zakhidov AA, Sachse C, Meiss J, Bikova J, Cook A, Zakhidov AA, Leo K (2012). Semi-transparent small molecule organic solar cells with laminated free-standing carbon nanotube top electrodes. Sol. Energy Mater. Sol. Cells.

[17] Zhang YD, Peng ZS, Cai CS, Liu Z, Lin YB, Zheng WH, Yang JY, Hou LT, Cao Y (2016). Colorful semitransparent polymer solar cells employing a bottom periodic one-dimensional photonic crystal and a top conductive PEDOT:PSS layer. J. Mater. Chem. A.

[18] Lunt RR, Bulovicl V (2011). Transparent, near-infrared organic photovoltaic solar cells for window and energy-scavenging applications. Appl. Phys. Lett..

[19] Long YB (2011). Optimizing one-dimensional photonic-crystals based semitransparent organic solar cells by tailoring reflection phase shift within photonic bandgap. Appl. Phys. Lett..

[20] Yu WJ, Shen L, Long YB, Guo WB, Meng FX, Ruan SP, Jia X, Ma HS, Chen WY (2012). Semitransparent polymer solar cells with one-dimensional (WO3/LiF)N photonic crystals. Appl. Phys. Lett..

[21] Yu WJ, Shen L, Shen P, Long YB, Sun HW, Chen WY, Ruan SP (2014). Semitransparent polymer solar cells with 5% power conversion efficiency using photonic crystal reflector. ACS Appl. Mater. Interfaces.

[22] Xu G, Shen L, Cui C, Wen S, Xue R, Chen W, Chen H, Zhang J, Li H, Li Y, Li Y (2017). High-performance colorful semitransparent polymer solar cells with ultrathin hybrid-metal electrodes and fine-tuned dielectric mirrors. Adv. Funct. Mater..

[23] Li XH, Choy WCH, Huo LJ, Xie FX, Sha WEI, Ding BF, Guo X, Li YF, Hou JH, You JB, Yang Y (2012). Dual plasmonic nanostructures for high performance inverted organic solar cells. Adv. Mater..

[24] Chen LM, Hong Z, Li G, Yang Y (2009). Recent progress in polymer solar cells: manipulation of polymer: Fullerene morphology and the formation of efficient inverted polymer solar cells. Adv. Mater..

[25] Long Y (2011). Improving optical performance of low bandgap polymer solar cells by the two-mode moderate microcavity. Appl. Phys. Lett..

[26] Lin HW, Chiu SW, Lin LY, Hung ZY, Chen YH, Lin F, Wong KT (2012). Device engineering for highly efficient top-illuminated organic solar cells with microcavity structures. Adv. Mater..

[27] Zhong J (2018). Optik – Int. J. Light Electron. Opt..

[28] Long Y (2009). Improving optical performance of inverted organic solar cells by microcavity effect. Appl. Phys. Lett..

[29] Lee J, Kim SY, Kim C, Kim JJ (2010). Enhancement of the short circuit current in organic photovoltaic devices with microcavity structures. Appl. Phys. Lett..

[30] Long Y (2011). Red and near-infrared absorption enhancement for low bandgap polymer solar cells by combining the optical microcavity and optical spacers. Sol. Energy Mater. Sol. Cells.

[31] Long Y (2010). Effects of metal electrode reflection and layer thicknesses on the performance of inverted organic solar cells. Sol. Energy Mater. Sol. Cells.

[32] Persson NK, Arwin H, Inganäs O (2005). Optical optimization of polyfluorene-fullerene blend photodiodes. J. Appl. Phys..

[33] Yang C, Liu D, Bates M, Barr MC, Lunt RR (2019). How to accurately report transparent solar cells. Joule.

[34] Traverse CJ, Pandey R, Barr MC, Lunt RR (2017). Emergence of highly transparent photovoltaics for distributed applications. Nat. Energy.

[35] Yu K, Song W, Ge J, Zheng K, Xie L, Chen Z, Qiu Yi, Hong L, Liu C, Ge Z (2022). 18.01% Efficiency organic solar cell and 2.53% light utilization efficiency semitransparent organic solar cell enabled by optimizing PM6: Y6 active layer morphology. Sci. China Chem..

[36] Almora O, Baran D, Bazan GC, Berger C, Cabrera CI, Catchpole KR, ErtenEla S (2021). Device performance of emerging photovoltaic materials (version 2). Adv. Energy Mater..

[37] Bendickson JM, Dowling JP, Scalora M (1996). Analytic expressions for the electromagnetic mode density in finite, one-dimensional, photonic band-gap structures. Phys. Rev. E.

[38] Macleod HA (2010). Thin-Film Optical Filters.

[39] Milani EA, Piralaee M, Ahmadi S, Asgari A (2022). The role of structural parameters on efficiency and transparency of semi-transparent non-fullerene organic solar cell. Sci. Rep..

